# Relationship between Plasma Levels of Zinc and Clinical Course of Pneumonia

**Published:** 2017

**Authors:** Parviz Saleh, Alireza Sadeghpour, Mohammad Mirza-Aghazadeh-Attari, Mohammad Hatampour, Mohammad Naghavi-Behzad, Aidin Tabrizi

**Affiliations:** 1 Chronic Renal Disease Referral Center, Tabriz University of Medical Sciences, Tabriz, Iran; 2 Department of Orthopedic Surgery, Tabriz University of Medical Sciences, Tabriz, Iran; 3 Medical Philosophy and History Research Center, Tabriz University of Medical Sciences, Tabriz, Iran; 4 Students’ Research Committee, Tabriz University of Medical Sciences, Tabriz, Iran; 5 Pediatric Health Research Center, Tabriz University of Medical Sciences, Tabriz, Iran

**Keywords:** Community-Acquired Infections, Pneumonia, Zinc

## Abstract

**Background:**

Pneumonia is a common disease and is more prevalent among children and the elderly. Zinc (Zn) is an essential substance for the human body and plays an important role in regulating the immune system. Studies have shown a possible relation between the Zn plasma levels and pneumonia.

**Materials and Methods:**

In a cross-sectional study, 100 patients with pneumonia, who were referred to the Educational-Medical Centers of Tabriz University of Medical Sciences, were included in the study. The plasma levels of Zn of all patients were measured. The patients were divided into two groups of normal and low plasma levels of Zn. The severity and clinical course of pneumonia, including the durations of fever, tachycardia, and tachypnea were evaluated and compared between the two groups.

**Results:**

The plasma levels of Zn were normal in 56 patients and low in 44 patients. The mean duration of fever, tachycardia, and tachypnea in the group with normal plasma levels of Zn were 1.58±0.68, 2.04±0.81, and 2.78±0.84 days, respectively; and those in the group with decreased Zn plasma levels were 1.72±0.70, 2.18±0.90, and 2.97±0.91 days, respectively. There were no statistically significant differences between the two groups (P>0.05). However, the incidence of severe pneumonia was significantly less in the group with normal Zn plasma levels (P=0.001).

**Conclusion:**

Based on the findings of the present study, there was no statistically significant relationship between the plasma levels of Zn and the clinical course of pneumonia. However, Zn lowered the incidence of severe pneumonia.

## INTRODUCTION

Pneumonia is an inflammatory state of the lungs, mainly affecting the alveoli, having diverse causative organisms and mechanisms (1). Pneumonia is one of the most common infectious diseases, affecting 4 to 6 million people in the United States of America in the form of community acquired pneumonia (CAD), more than a million of those affected requiring hospitalization (2, 3). The most common age groups involved are children younger than 5 years of age and elderly aged more than 65 years (4, 5). Risk factors for acquiring pneumonia include age more than 60 years, alcoholism, cardiac diseases, immunosuppressive therapies, smoking, history of previous pneumonia, mechanical ventilation, body mass index (BMI) less than 18.5, and diabetes (6–10). Pneumonia imposes a heavy burden on the health system of nations worldwide with an estimated expenditure of 17 billion dollars in the United States alone (11); this is more disturbing, knowing that the disease is almost 5 times more common in the developing countries, causing catastrophic health expenditures in nations most vulnerable to infectious diseases (12).

The immune system requires different macro- and micro-nutrients to function efficiently (13), one of the most important being Zinc (Zn). There have been studies suggesting that Zn supplementation might have a beneficial effect in preventing infectious diseases (14), regulating the immune system, and enhancing the cellular immunity (15), being an essential factor for the optimal functioning of macrophages and lymphocytes (16). Moreover, it has been shown that Zn could function as an anti-oxidant (17), playing an important role in the course of infectious diseases. It has also been demonstrated that children with low levels of plasma Zn were at a higher risk of acquiring pneumonia (18, 19). It has also been suggested that supplementation of Zn in the therapeutic regimen of children with pneumonia could improve the results, and boost the chances of leaving the hospital with no long term complications (20). However, data on the significance of Zn in preventing and treating pneumonia are limited and inconclusive (21). A study found that supplementation of Zn in children with severe pneumonia did not have any significant effect on the duration of the disease (22). Moreover, Zn plasma levels are confounded by several aspects, including geographical factors, race, sex, and socio-economic status (23–25), making it harder to generalize the results of these studies to different contexts. There are also limited studies on the plasma levels of Zn in patients with pneumonia, analyzing the relation between it and the clinical outcome.

The present study sought to further evaluate the effect of plasma levels of Zn on the clinical course of patients with pneumonia, and the relation between the severity of the disease acquired and plasma levels of Zn.

## MATERIALS AND METHODS

During the present cross-sectional study, conducted between April 2015 and April 2016, in the Educational-Medical Centers of Tabriz University of Medical Sciences (Tabriz, Iran), these being the main referral centers for infectious diseases in North-Western Iran. A total of 100 patients were included in this study; they were divided into two groups with low (44 patients) and normal (56 patients) plasma zinc levels. The age, sex, and risk factors, such as smoking, occupation, and BMI, were evaluated; the differences between the two groups were not significant. A drug history was obtained from all patients before inclusion in the study. The inclusion criteria were age between 15 and 65 years, and a definite diagnosis of pneumonia on chest radiography, the gold-standard test for the diagnosis of pneumonia (26, 27). The exclusion criteria were hemodynamic instability, presence of congenital respiratory defects, history of previous autoimmune diseases, pneumonia caused by medication, chemotherapy, hospitalization during the previous year, and use of Zn supplementation during the two months prior to inclusion in the study. All patients were clearly informed about the steps of the study and written informed consent was obtained from all patients; the patients were treated using the most recent guidelines, and after the disease was cured, they were discharged from the infectious disease ward. The study protocol was approved by the Regional Ethics Committee of Tabriz University of Medical Sciences, and was compliant with the tenets of the Declaration of Helsinki. In the present study, an extra 2 cc of blood was obtained from the patients during the tests ordered by the attending specialists in the infectious disease ward; these samples were collected in previously heparinized Eppendoff test tubes and sent to the central laboratory of Tabriz University of Medical Sciences to determine the plasma levels of Zn using a Hitachi 902 biochemistry analyzer and the common kits available. Plasma levels of Zn more than 60 mg/mL were considered normal (28) and those less than 60 mg/mL were considered low; moreover, the age, sex, severity of pneumonia and the number of days hospitalized, number of days with fever, tachycardia, and tachypnea, and possible mortality were determined for each patient and the means of the aforementioned variables were compared between the two groups. The severity of pneumonia was determined using the pneumonia severity index (PSI) or PORT Score (29). Statistical analysis was performed using the Statistical Package for the Social Sciences (SPSS) software version 16.0 (SPSS Inc., Chicago, IL). Quantitative data were presented as mean ± standard deviation (SD) and qualitative data as frequency and percentages (%). For statistical analysis, after determining the distribution of continuous variables using the Klomogorov-Smirnov test, independent sample t-test was used to compare the results between the two groups. Moreover, the collected data were analyzed using descriptive statistical methods, mean difference test for independent groups, and chi-square test or Fisher’s exact test. A P-value less than 0.05 was considered statistically significant in all steps.

## RESULTS

The mean age was 36.19±14.83 years (range, 16–65 years). The mean age of patients with normal Zn levels was 37.08±16.16 years, and the mean for the group Zn levels was 35.04±13.05 years, the difference was not statistically significant (P=0.497). A total of 56 patients had normal plasma levels of Zn and 44 patients had low plasma levels of Zn. The mean plasma level of Zn in patients with normal Zn levels was 71.08±8.32 mg/mL, and the mean for the other group was 14.36±6.98 mg/mL; the difference was statistically significant (P=0.001). Of the 56 patients with normal plasma levels of Zn, 27 (48.2%) were men and 29 (51.8%) were women; of the 44 with low Zn levels, 20 (45.5%) were men and 24 (54.5%) were women; the difference between the two groups was not statistically significant (P=0.842).

The mean number of days hospitalized and the mean number of days with leukocytosis, tachycardia, tachypnea, and fever in the 100 patients included in the study are summarized in Table 1. The means of the above-mentioned variables for the two groups and the comparison between them are shown in Table 2. The differences between the two groups regarding the number of days of hospitalization and the number of days with leukocytosis, tachycardia, tachypnea, and fever were not statistically significant (P>0.05). During the present study, no case of mortality was observed, and all patients were discharged from the infectious disease ward; thus, the groups had no significant differences between them.

**Table 1.** Variables in patients being included

**Variables**	**Amount of the variable**
Age	37.08±16.16
Days of Hospitalization	6.54±1.40
Duration of Leukocytosis (Days)	6.05±1.11
Duration of Tachypnea (Days)	2.89±0.90
Duration of Tachycardia (Days)	2.12±0.85
Duration of Fever (Days)	1.65±0.72
Percentage of patients with severe pneumonia	28% (28 out of 100)

**Table 2.** Comparison of clinical course of patients in the two groups of study

**Variables**	**Groups**		**P-value**
	**Normal plasma zinc level**	**Low plasma zinc level**	
Days of Hospitalization	6.43±1.38	6.61±1.50	0.358
Duration of Leukocytosis (Days)	5.96±1.14	6.20±1.06	0.548
Duration of Tachypnea (Days)	2.78±0.84	2.97±0.91	0.438
Duration of Tachycardia (Days)	2.04±0.81	2.18±0.90	0.267
Duration of Fever (Days)	1.580±.68	1.72±0.70	0.206
Percentage of patients with severe pneumonia	3.5% (2 out of 56)	59% (26 out of 44)	0.001

Data was shown as mean ± standard deviation

Regarding the severity of acquired pneumonia as determined by the PSI score, of the total of 100 patients, 28 had severe pneumonia, 26 belonging to the group with low plasma levels of Zn (59%), and the remaining two belonging to the group with normal plasma levels of Zn (3.5%); the difference between the two groups was statistically significant (P=0.001). The data are summarized in Table 1.

## DISCUSSION

During the present cross-sectional study, it was found that there was a significant relation between the Zn plasma levels and the severity of the disease acquired (P=0.001). However, there was no significant relation between the Zn plasma levels and the clinical course of pneumonia (days of hospitalization [P=0.358], duration of leukocytosis [P=0.548], duration of tachypnea [P=0.438], duration of tachycardia [P=0.267], and duration of fever [P=0.206]).

In different studies by Osendarp et al. and Baqui et al., it was demonstrated that children who used Zn supplements had less morbidity due to respiratory tract infections; the present study did not show any relation between the Zn levels and the morbidity due to pneumonia (30, 31).

A cross-sectional study by Arica et al. showed that there was a significant relation between the plasma levels of Zn and the susceptibility to pneumonia in children aged 0–24 months (32), which was in contrast to the present study. However, the difference in the results might have been caused by the different age groups considered, the present one having a wide range (16–65 years); moreover, Zn may be more protective in children, whose immune systems are not fully developed.

In another study by Brooks et al., it was shown that there was a significant relation between the declining rates of severe pneumonia and the use of Zn supplementation, which is in line with the present study. However, Brooks et al. also reported a decline in the symptoms of non-severe pneumonia after Zn supplementation (18); this was not in line with the present study, which showed no relation between the plasma levels of Zn and the clinical course of the disease.

Meydani et al. conducted a study to examine the effect of Zn supplementation on the incidence of pneumonia among the residents of an elderly home. They found out that the incidence of pneumonia declined significantly, and the need for antibiotic therapy was reduced after Zn supplementation; this was in contrast to the present study (33).

Barnett et al., in an observational study, found that Zn supplementation and having adequate plasma levels of Zn could have a beneficial effect on the clinical course and incidence of pneumonia (34); however, they also cited that more evidence was needed to fully accept the findings.

In different studies conducted by Pushpa and Memon (35), and Kumar et al. (36), it was shown that children with normal plasma levels of Zn were in less danger of acquiring severe pneumonia; this was in line with the present study.

Vinayak and Behal found that a course of Zn supplementation for patients with pneumonia, aged up to 5 years, did not have a significant effect and recommended that it should not be proposed for adjuvant therapies (37); this was in line with the present study results. However, Zhou et al. and Lassi et al. reported the opposite findings in younger patients, claiming that high levels of Zn in pediatric patients with pneumonia could lower the incidence and prevalence of pneumonia, days of hospitalization, and improve the clinical outcome (38, 39). It appears that the significance of plasma levels of Zn and its supplementation depends on the age of the patient, younger patients being better candidates for Zn supplementation.

In the future, researches comparing immunosuppressed patients and those with intact immunity are required. Further studies are also needed to completely clarify the relation between the significance of the possible positive effect of Zn in pneumonia with age, and the preventive effect of Zn in pneumonia. A clinical trial examining the supplementation of Zn in patients with low Zn levels and the subsequent change in the clinical course, would be of great benefit.

## CONCLUSION

There is a significant relation between the risk of severe pneumonia and plasma levels of Zn, with patients having normal Zn levels being in less danger of severe pneumonia, although there is no significant relation between the plasma levels of Zn in patients with pneumonia and the clinical course of the disease.
